# 1,2-Bis(*p*-tolyl­sulfon­yl)hydrazine

**DOI:** 10.1107/S1600536808034314

**Published:** 2008-10-25

**Authors:** Wei-Fen Zhang, Ruo-Bao Li, Xi-Shi Tai, Shu-Hong Tang, Jin-Bao Tang

**Affiliations:** a Department of Basic Medicine, Weifang Medical University, Weifang 261053, People’s Republic of China; b Department of Chemistry and Chemical Engineering, Weifang University, Weifang 261061, People’s Republic of China; c College of Information and Control Engineering, Weifang University, Weifang 261061, People’s Republic of China

## Abstract

In the title compound, C_14_H_16_N_2_O_4_S_2_, the dihedral angle between the aromatic ring planes is 76.8 (3)° and the S—N—N—S torsion angle is 122.5 (3)°. In the crystal structure, mol­ecules form a chain structure by way of N—H⋯O hydrogen bonds.

## Related literature

For background on aroylhydrazines, see: Bu *et al.* (2001 ▶); Ranford *et al.* (1998 ▶), Agarwal & Sharma (1993 ▶).
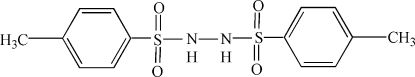


## Experimental

### 

#### Crystal data

C_14_H_16_N_2_O_4_S_2_
*M* *_r_* = 340.41
Monoclinic, 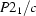

*a* = 15.7318 (16) Å
*b* = 10.7016 (12) Å
*c* = 9.4943 (9) Å
β = 90.102 (2)°
*V* = 1598.4 (3) Å^3^
*Z* = 4
Mo *K*α radiation
μ = 0.35 mm^−1^
*T* = 298 (2) K
0.33 × 0.11 × 0.04 mm

#### Data collection

Bruker SMART CCD diffractometer
Absorption correction: multi-scan (*SADABS*; Bruker, 2000 ▶) *T*_min_ = 0.893, *T*_max_ = 0.986
7984 measured reflections
2808 independent reflections
1132 reflections with *I* > 2σ(*I*)
*R*_int_ = 0.108

#### Refinement

*R*[*F* ^2^ > 2σ(*F* ^2^)] = 0.065
*wR*(*F* ^2^) = 0.080
*S* = 0.97
2808 reflections
199 parameters
H-atom parameters constrained
Δρ_max_ = 0.23 e Å^−3^
Δρ_min_ = −0.27 e Å^−3^

### 

Data collection: *SMART* (Bruker, 2000 ▶); cell refinement: *SAINT* (Bruker, 2000 ▶); data reduction: *SAINT*; program(s) used to solve structure: *SHELXS97* (Sheldrick, 2008 ▶); program(s) used to refine structure: *SHELXL97* (Sheldrick, 2008 ▶); molecular graphics: *SHELXTL* (Sheldrick, 2008 ▶); software used to prepare material for publication: *SHELXTL*.

## Supplementary Material

Crystal structure: contains datablocks global, I. DOI: 10.1107/S1600536808034314/hb2824sup1.cif

Structure factors: contains datablocks I. DOI: 10.1107/S1600536808034314/hb2824Isup2.hkl

Additional supplementary materials:  crystallographic information; 3D view; checkCIF report

## Figures and Tables

**Table 1:** Hydrogen-bond geometry (Å, °)

*D*—H⋯*A*	*D*—H	H⋯*A*	*D*⋯*A*	*D*—H⋯*A*
N1—H1⋯O3^i^	0.90	2.10	2.935 (5)	155
N2—H2*A*⋯O1^i^	0.90	2.01	2.857 (5)	157

Symmetry code: (i) 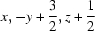.

**Fig. 1.**
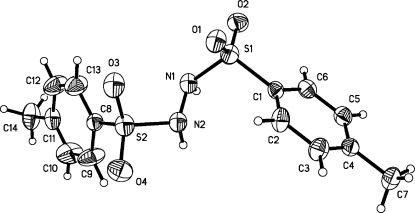
The molecular structure of (I) showing 30% displacement ellipsoids (arbitrary spheres for the H atoms).

## supplementary crystallographic information

### Comment

Aroylhydrazines have drawn much attention in recent years due to their wide biological and pharmacological activities, such as antitumor (Ranford *et al.*, 1998), antidiabetic (Bu *et al.*, 2001) antitubercular (Agarwal & Sharma, 1993) activities. As part of our studies in this area, we now report the synthesis and crystal structure of the title compound, (I).

In (I), the dihedral angle between the two phenyl rings of (C1—C6 and C8—C13) is 76.8 (3)°. The S=O bond distances are characteristic of double bonds. The N1—N2 single bond of 1.407 (4) is in agreement with that of other acylhydrazone compounds in which the acylhydrazone takes a ketonic form.

In the crystal, N—H···O hydrogen bonds (Table 1) help to establish the packing.

### Experimental

5 mmol of 4-toluene sulfonyl chloride (5 mmol) was added to a solution of hydrazine (2.5 mmol) in 10 ml of ethanol at room temperature. The mixture was continuously stirred for 2 h at temperature, the solid product was collected by filtration and dried *in vacuo* (yield 58%). Clear blocks of (I) were obtained by evaporation from a methanol/water solution after 10 days.

### Crystal data

**Table:**

C_14_H_16_N_2_O_4_S_2_	*F*(000) = 712
*M**_r_* = 340.41	*D*_x_ = 1.415 Mg m^−^^3^
Monoclinic, *P*2_1_/*c*	Mo *K*α radiation, λ = 0.71073 Å
Hall symbol: -P 2ybc	Cell parameters from 586 reflections
*a* = 15.7318 (16) Å	θ = 2.3–25.0°
*b* = 10.7016 (12) Å	µ = 0.35 mm^−^^1^
*c* = 9.4943 (9) Å	*T* = 298 K
β = 90.102 (2)°	Flake, colourless
*V* = 1598.4 (3) Å^3^	0.33 × 0.11 × 0.04 mm
*Z* = 4	

### Data collection

**Table:**

Bruker SMART CCD diffractometer	2808 independent reflections
Radiation source: fine-focus sealed tube	1132 reflections with *I* > 2σ(*I*)
graphite	*R*_int_ = 0.108
ω scans	θ_max_ = 25.0°, θ_min_ = 2.3°
Absorption correction: multi-scan (SADABS; Bruker, 2000)	*h* = −18→12
*T*_min_ = 0.893, *T*_max_ = 0.986	*k* = −12→12
7984 measured reflections	*l* = −11→11

### Refinement

**Table:**

Refinement on *F*^2^	Primary atom site location: structure-invariant direct methods
Least-squares matrix: full	Secondary atom site location: difference Fourier map
*R*[*F*^2^ > 2σ(*F*^2^)] = 0.065	Hydrogen site location: inferred from neighbouring sites
*wR*(*F*^2^) = 0.080	H-atom parameters constrained
*S* = 0.97	*w* = 1/[σ^2^(*F*_o_^2^) + (0.0003*P*)^2^] where *P* = (*F*_o_^2^ + 2*F*_c_^2^)/3
2808 reflections	(Δ/σ)_max_ < 0.001
199 parameters	Δρ_max_ = 0.23 e Å^−^^3^
0 restraints	Δρ_min_ = −0.27 e Å^−^^3^

### Special details

**Table:**

Geometry. All e.s.d.'s (except the e.s.d. in the dihedral angle between two l.s. planes) are estimated using the full covariance matrix. The cell e.s.d.'s are taken into account individually in the estimation of e.s.d.'s in distances, angles and torsion angles; correlations between e.s.d.'s in cell parameters are only used when they are defined by crystal symmetry. An approximate (isotropic) treatment of cell e.s.d.'s is used for estimating e.s.d.'s involving l.s. planes.
Refinement. Refinement of *F*^2^ against ALL reflections. The weighted *R*-factor *wR* and goodness of fit *S* are based on *F*^2^, conventional *R*-factors *R* are based on *F*, with *F* set to zero for negative *F*^2^. The threshold expression of *F*^2^ > σ(*F*^2^) is used only for calculating *R*-factors(gt) *etc*. and is not relevant to the choice of reflections for refinement. *R*-factors based on *F*^2^ are statistically about twice as large as those based on *F*, and *R*- factors based on ALL data will be even larger.

### Fractional atomic coordinates and isotropic or equivalent isotropic displacement parameters (Å^2^)

**Table:**

	*x*	*y*	*z*	*U*_iso_*/*U*_eq_	
S1	0.82111 (9)	0.60680 (14)	−0.00482 (14)	0.0542 (4)	
S2	0.70156 (9)	0.91574 (15)	−0.01299 (15)	0.0588 (4)	
N1	0.7517 (2)	0.7008 (4)	0.0725 (4)	0.0513 (12)	
H1	0.7484	0.6794	0.1640	0.062*	
N2	0.7743 (2)	0.8278 (4)	0.0630 (4)	0.0535 (12)	
H2A	0.7823	0.8571	0.1508	0.064*	
O1	0.82411 (18)	0.6447 (3)	−0.1491 (3)	0.0628 (10)	
O2	0.7938 (2)	0.4847 (3)	0.0334 (4)	0.0682 (11)	
O3	0.6846 (2)	0.8623 (3)	−0.1482 (3)	0.0701 (11)	
O4	0.7356 (2)	1.0395 (3)	−0.0015 (4)	0.0771 (13)	
C1	0.9213 (3)	0.6382 (5)	0.0667 (5)	0.0446 (14)	
C2	0.9673 (4)	0.7381 (5)	0.0188 (6)	0.0602 (17)	
H2	0.9458	0.7892	−0.0520	0.072*	
C3	1.0465 (4)	0.7620 (5)	0.0775 (6)	0.0634 (17)	
H3	1.0772	0.8310	0.0467	0.076*	
C4	1.0807 (3)	0.6865 (5)	0.1799 (6)	0.0534 (16)	
C5	1.0339 (4)	0.5857 (5)	0.2236 (5)	0.0579 (15)	
H5	1.0567	0.5327	0.2915	0.069*	
C6	0.9547 (3)	0.5604 (5)	0.1703 (6)	0.0538 (15)	
H6	0.9238	0.4923	0.2030	0.065*	
C7	1.1679 (3)	0.7141 (5)	0.2376 (6)	0.080 (2)	
H7A	1.1862	0.6461	0.2962	0.121*	
H7B	1.1661	0.7895	0.2924	0.121*	
H7C	1.2071	0.7245	0.1611	0.121*	
C8	0.6086 (3)	0.9042 (5)	0.0880 (5)	0.0472 (14)	
C9	0.6008 (4)	0.9722 (5)	0.2078 (6)	0.087 (2)	
H9	0.6448	1.0240	0.2374	0.104*	
C10	0.5265 (4)	0.9636 (6)	0.2860 (6)	0.095 (2)	
H10	0.5214	1.0115	0.3673	0.115*	
C11	0.4622 (4)	0.8893 (6)	0.2488 (6)	0.0659 (18)	
C12	0.4712 (4)	0.8228 (5)	0.1302 (7)	0.087 (2)	
H12	0.4274	0.7698	0.1025	0.104*	
C13	0.5441 (4)	0.8306 (5)	0.0467 (6)	0.083 (2)	
H13	0.5480	0.7851	−0.0365	0.100*	
C14	0.3808 (3)	0.8797 (5)	0.3358 (6)	0.091 (2)	
H14A	0.3852	0.9327	0.4170	0.136*	
H14B	0.3727	0.7947	0.3654	0.136*	
H14C	0.3332	0.9055	0.2794	0.136*	

### Atomic displacement parameters (Å^2^)

**Table:**

	*U*^11^	*U*^22^	*U*^33^	*U*^12^	*U*^13^	*U*^23^
S1	0.0498 (10)	0.0731 (11)	0.0398 (9)	0.0048 (9)	0.0043 (7)	−0.0049 (8)
S2	0.0622 (11)	0.0726 (11)	0.0417 (10)	0.0093 (10)	0.0038 (8)	0.0035 (9)
N1	0.052 (3)	0.068 (3)	0.034 (3)	0.010 (3)	0.004 (2)	0.002 (2)
N2	0.054 (3)	0.068 (3)	0.038 (3)	0.010 (3)	−0.001 (2)	−0.003 (3)
O1	0.058 (2)	0.099 (3)	0.031 (2)	0.011 (2)	0.0041 (17)	−0.007 (2)
O2	0.066 (3)	0.056 (2)	0.083 (3)	−0.009 (2)	0.003 (2)	−0.001 (2)
O3	0.073 (3)	0.104 (3)	0.033 (2)	0.021 (2)	0.0053 (19)	−0.004 (2)
O4	0.087 (3)	0.062 (2)	0.082 (3)	−0.014 (2)	0.012 (2)	0.010 (2)
C1	0.038 (3)	0.051 (4)	0.045 (4)	0.006 (3)	0.006 (3)	−0.005 (3)
C2	0.048 (4)	0.071 (4)	0.061 (4)	0.005 (4)	0.004 (3)	0.022 (3)
C3	0.048 (4)	0.076 (5)	0.066 (5)	−0.007 (4)	0.011 (3)	0.008 (4)
C4	0.051 (4)	0.058 (4)	0.051 (4)	0.007 (4)	−0.003 (3)	−0.013 (3)
C5	0.067 (4)	0.058 (4)	0.049 (4)	0.014 (4)	−0.012 (3)	0.002 (3)
C6	0.058 (4)	0.056 (4)	0.047 (4)	0.004 (3)	0.009 (3)	−0.001 (3)
C7	0.052 (4)	0.096 (5)	0.093 (6)	0.005 (4)	−0.012 (4)	−0.017 (4)
C8	0.049 (4)	0.055 (4)	0.038 (3)	0.006 (3)	−0.002 (3)	−0.003 (3)
C9	0.081 (5)	0.112 (5)	0.067 (5)	−0.030 (4)	0.015 (4)	−0.043 (4)
C10	0.094 (6)	0.124 (6)	0.069 (5)	−0.008 (5)	0.034 (4)	−0.042 (4)
C11	0.057 (4)	0.082 (5)	0.058 (4)	0.020 (4)	0.005 (4)	0.010 (4)
C12	0.057 (5)	0.112 (5)	0.092 (6)	−0.024 (4)	0.007 (4)	−0.029 (5)
C13	0.071 (5)	0.103 (5)	0.075 (5)	−0.003 (4)	0.001 (4)	−0.031 (4)
C14	0.068 (4)	0.132 (5)	0.072 (5)	0.026 (4)	0.017 (3)	0.020 (4)

### Geometric parameters (Å, °)

**Table:**

S1—O2	1.423 (3)	C5—H5	0.9300
S1—O1	1.429 (3)	C6—H6	0.9300
S1—N1	1.657 (3)	C7—H7A	0.9600
S1—C1	1.748 (5)	C7—H7B	0.9600
S2—O3	1.430 (3)	C7—H7C	0.9600
S2—O4	1.433 (3)	C8—C13	1.343 (6)
S2—N2	1.648 (4)	C8—C9	1.356 (6)
S2—C8	1.754 (4)	C9—C10	1.389 (6)
N1—N2	1.407 (4)	C9—H9	0.9300
N1—H1	0.9001	C10—C11	1.333 (7)
N2—H2A	0.8998	C10—H10	0.9300
C1—C2	1.369 (6)	C11—C12	1.340 (7)
C1—C6	1.391 (6)	C11—C14	1.529 (6)
C2—C3	1.387 (7)	C12—C13	1.398 (6)
C2—H2	0.9300	C12—H12	0.9300
C3—C4	1.373 (7)	C13—H13	0.9300
C3—H3	0.9300	C14—H14A	0.9600
C4—C5	1.370 (6)	C14—H14B	0.9600
C4—C7	1.507 (6)	C14—H14C	0.9600
C5—C6	1.371 (6)		
			
O2—S1—O1	121.0 (2)	C5—C6—C1	119.0 (5)
O2—S1—N1	104.1 (2)	C5—C6—H6	120.5
O1—S1—N1	106.0 (2)	C1—C6—H6	120.5
O2—S1—C1	110.5 (3)	C4—C7—H7A	109.5
O1—S1—C1	106.6 (2)	C4—C7—H7B	109.5
N1—S1—C1	107.8 (2)	H7A—C7—H7B	109.5
O3—S2—O4	120.5 (2)	C4—C7—H7C	109.5
O3—S2—N2	107.0 (2)	H7A—C7—H7C	109.5
O4—S2—N2	103.6 (2)	H7B—C7—H7C	109.5
O3—S2—C8	107.9 (2)	C13—C8—C9	119.3 (5)
O4—S2—C8	109.6 (2)	C13—C8—S2	120.8 (4)
N2—S2—C8	107.4 (2)	C9—C8—S2	119.8 (5)
N2—N1—S1	113.0 (3)	C8—C9—C10	119.4 (6)
N2—N1—H1	108.8	C8—C9—H9	120.3
S1—N1—H1	108.2	C10—C9—H9	120.3
N1—N2—S2	113.8 (3)	C11—C10—C9	122.5 (6)
N1—N2—H2A	108.2	C11—C10—H10	118.8
S2—N2—H2A	107.5	C9—C10—H10	118.8
C2—C1—C6	120.2 (5)	C10—C11—C12	117.3 (6)
C2—C1—S1	119.9 (5)	C10—C11—C14	122.2 (6)
C6—C1—S1	119.9 (4)	C12—C11—C14	120.5 (7)
C1—C2—C3	119.1 (5)	C11—C12—C13	122.2 (6)
C1—C2—H2	120.5	C11—C12—H12	118.9
C3—C2—H2	120.5	C13—C12—H12	118.9
C4—C3—C2	121.7 (5)	C8—C13—C12	119.3 (6)
C4—C3—H3	119.1	C8—C13—H13	120.3
C2—C3—H3	119.1	C12—C13—H13	120.3
C5—C4—C3	117.9 (6)	C11—C14—H14A	109.5
C5—C4—C7	122.2 (6)	C11—C14—H14B	109.5
C3—C4—C7	119.8 (6)	H14A—C14—H14B	109.5
C4—C5—C6	122.1 (5)	C11—C14—H14C	109.5
C4—C5—H5	119.0	H14A—C14—H14C	109.5
C6—C5—H5	119.0	H14B—C14—H14C	109.5
			
O2—S1—N1—N2	172.0 (3)	C4—C5—C6—C1	1.3 (8)
O1—S1—N1—N2	−59.3 (4)	C2—C1—C6—C5	0.2 (7)
C1—S1—N1—N2	54.6 (4)	S1—C1—C6—C5	179.1 (4)
S1—N1—N2—S2	122.5 (3)	O3—S2—C8—C13	13.6 (5)
O3—S2—N2—N1	−55.6 (3)	O4—S2—C8—C13	146.6 (4)
O4—S2—N2—N1	176.0 (3)	N2—S2—C8—C13	−101.5 (5)
C8—S2—N2—N1	60.1 (4)	O3—S2—C8—C9	−165.1 (4)
O2—S1—C1—C2	165.9 (4)	O4—S2—C8—C9	−32.1 (5)
O1—S1—C1—C2	32.5 (4)	N2—S2—C8—C9	79.8 (5)
N1—S1—C1—C2	−80.9 (4)	C13—C8—C9—C10	0.4 (9)
O2—S1—C1—C6	−13.1 (4)	S2—C8—C9—C10	179.1 (5)
O1—S1—C1—C6	−146.4 (4)	C8—C9—C10—C11	1.0 (10)
N1—S1—C1—C6	100.2 (4)	C9—C10—C11—C12	−0.9 (10)
C6—C1—C2—C3	−1.5 (8)	C9—C10—C11—C14	179.6 (6)
S1—C1—C2—C3	179.6 (4)	C10—C11—C12—C13	−0.5 (10)
C1—C2—C3—C4	1.5 (9)	C14—C11—C12—C13	179.0 (5)
C2—C3—C4—C5	−0.2 (8)	C9—C8—C13—C12	−1.7 (8)
C2—C3—C4—C7	178.3 (5)	S2—C8—C13—C12	179.5 (4)
C3—C4—C5—C6	−1.2 (8)	C11—C12—C13—C8	1.9 (9)
C7—C4—C5—C6	−179.7 (5)		

### Hydrogen-bond geometry (Å, °)

**Table:**

*D*—H···*A*	*D*—H	H···*A*	*D*···*A*	*D*—H···*A*
N1—H1···O3^i^	0.90	2.10	2.935 (5)	155
N2—H2A···O1^i^	0.90	2.01	2.857 (5)	157

Symmetry codes: (i) *x*, −*y*+3/2, *z*+1/2.
